# [(2*R*,3*S*,6*S*)-3-Acet­yloxy-6-(1-phenyl-1*H*-1,2,3-triazol-4-yl)-3,6-dihydro-2*H*-pyran-2-yl]methyl acetate

**DOI:** 10.1107/S1600536811037305

**Published:** 2011-09-30

**Authors:** Julio Zukerman-Schpector, Hélio A. Stefani, Nathalia C. S. Silva, Seik Weng Ng, Edward R. T. Tiekink

**Affiliations:** a Department of Chemistry, Universidade Federal de São Carlos, 13565-905 São Carlos, SP, Brazil; b Departamento de Farmácia, Faculdade de Ciências Farmacêuticas, Universidade de São Paulo, São Paulo-SP, Brazil; c Department of Chemistry, University of Malaya, 50603 Kuala Lumpur, Malaysia; d Chemistry Department, Faculty of Science, King Abdulaziz University, PO Box 80203 Jeddah, Saudi Arabia

## Abstract

In the title compound, C_18_H_19_N_3_O_5_, the 3,6-dihydro-2*H*-pyran ring adopts a half-chair, distorted towards a half-boat, conformation with *Q*_T_ = 0.5276(14) Å. The benzene ring is twisted out of the place of the triazole ring [dihedral angle = 23.54 (8)°]. In the crystal, supra­molecular layers in the *ac* plane are formed through C—H⋯O and C—H⋯π(triazole) inter­actions. These stack along the *b* axis being connected by C—H⋯N contacts.

## Related literature

For background to the chemical attributes of *C*-glycosides, see: Ritchie *et al.* (2002 ▶); Hanessian & Lou (2000 ▶); Hultin (2005 ▶); Zou (2005 ▶). For chiral properties of *C*-glycosides, see: Nakata (2005 ▶); Nicolaou *et al.* (2008 ▶); Somsak (2001 ▶). For additional conformation analysis, see: Cremer & Pople (1975 ▶).
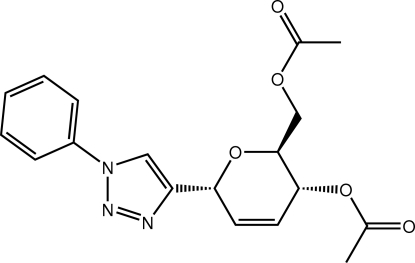


## Experimental

### 

#### Crystal data

C_18_H_19_N_3_O_5_
*M* *_r_* = 357.36
Monoclinic, 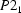

*a* = 4.79932 (7) Å
*b* = 16.6308 (2) Å
*c* = 10.76331 (14) Å
β = 93.225 (1)°
*V* = 857.73 (2) Å^3^
*Z* = 2
Cu *K*α radiation
μ = 0.86 mm^−1^
*T* = 100 K
0.20 × 0.10 × 0.05 mm

#### Data collection

Agilent SuperNova Dual Cu at zero diffractometer with an Atlas detector
Absorption correction: multi-scan (*CrysAlis PRO*; Agilent, 2010 ▶) *T*_min_ = 0.848, *T*_max_ = 0.959
5784 measured reflections
3369 independent reflections
3304 reflections with *I* > 2σ(*I*)
*R*_int_ = 0.019

#### Refinement

*R*[*F* ^2^ > 2σ(*F* ^2^)] = 0.033
*wR*(*F* ^2^) = 0.084
*S* = 1.04
3369 reflections
237 parameters
1 restraint
H-atom parameters constrained
Δρ_max_ = 0.14 e Å^−3^
Δρ_min_ = −0.19 e Å^−3^
Absolute structure: Flack (1983 ▶), 1591 Friedel pairs
Flack parameter: −0.09 (15)

### 

Data collection: *CrysAlis PRO* (Agilent, 2010 ▶); cell refinement: *CrysAlis PRO*; data reduction: *CrysAlis PRO*; program(s) used to solve structure: *SIR92* (Altomare *et al.*, 1999 ▶); program(s) used to refine structure: *SHELXL97* (Sheldrick, 2008 ▶); molecular graphics: *ORTEP-3* (Farrugia, 1997 ▶), *DIAMOND* (Brandenburg, 2006 ▶) and *MarvinSketch* (ChemAxon, 2009 ▶); software used to prepare material for publication: *publCIF* (Westrip, 2010 ▶).

## Supplementary Material

Crystal structure: contains datablock(s) global, I. DOI: 10.1107/S1600536811037305/hg5093sup1.cif

Structure factors: contains datablock(s) I. DOI: 10.1107/S1600536811037305/hg5093Isup2.hkl

Supplementary material file. DOI: 10.1107/S1600536811037305/hg5093Isup3.cml

Additional supplementary materials:  crystallographic information; 3D view; checkCIF report

## Figures and Tables

**Table 1:** Hydrogen-bond geometry (Å, °)

*D*—H⋯*A*	*D*—H	H⋯*A*	*D*⋯*A*	*D*—H⋯*A*
C7—H7⋯O4^i^	0.95	2.29	3.2207 (19)	167
C9—H9⋯*Cg*1^ii^	1.00	2.68	3.5362 (16)	144
C16—H16a⋯N3^iii^	0.98	2.62	3.463 (2)	145
C16—H16b⋯O2^ii^	0.98	2.59	3.570 (2)	177
C18—H18a⋯O1^iv^	0.98	2.54	3.516 (2)	174
C18—H18c⋯O4^v^	0.98	2.45	3.400 (2)	164

Symmetry codes: (i) 
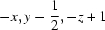
; (ii) 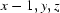; (iii) 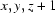; (iv) 
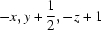
; (v) 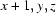.

**Fig. 1.**
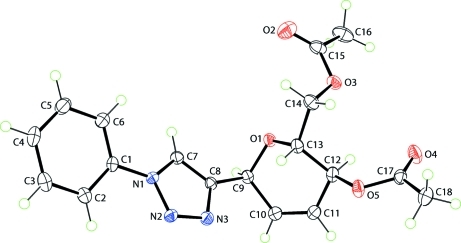
The molecular structure of compound (I) showing atom labelling scheme and displacement ellipsoids at the 50% probability level (arbitrary spheres for the H atoms).

**Fig. 2.**
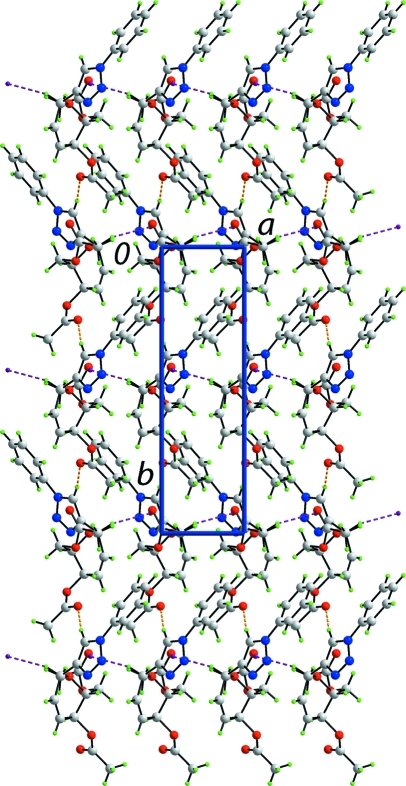
A view in projection down the c axis showing the supramolecular array sustained by relatviely strong C—H···O contacts (orange dashed lines) formed along the b direction and C—H···π contacts (purple dashed lines) formed along the a direction.

**Fig. 3.**
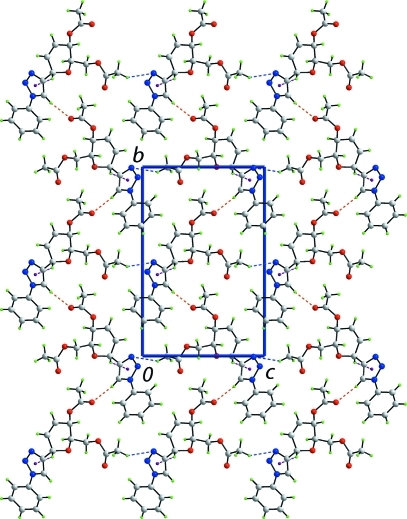
A view in projection down the a axis highlighting the stacking of zigzag layers along the b direction. The C—H···O, C—H···π and C—H···N interactions are shown as orange, purple and blue dashed lines, respectively.

## supplementary crystallographic information

### Comment

The chemistry and biological activity of *C*-glycosides has experienced increased attention due to their structural similarity to carbohydrates but also due to their resistance to metabolic processes. Such attributes may lead to improved biological profiles as compared to their *O*-analogues (Ritchie *et al.* 2002; Hanessian & Lou, 2000; Hultin, 2005; Zou, 2005). In addition, *C*-glycosides have also been found embedded in the structure of several bioactive natural products (Nakata, 2005; Nicolaou *et al.* 2008), and served as chiral building blocks for the stereoselective synthesis of optically active compounds (Somsak, 2001).

The title compound, (I), Fig. 1, was prepared in connection with on-going research into the synthesis of *C*-glycosides. The absolute structure was confirmed experimentally and shows the chirality at the C9, C12 and C13 atoms to be *S*, *S*, and *R*, respectively. The dihedral angle between the phenyl and the triazole ring is 23.54 (8) °. The 3,6-dihydro-2*H*-pyran ring has a distorted half-chair conformation with the O1 atom lying 0.6127 (16) Å above the plane defined by the C9–C13 atoms (r.m.s. deviation = 0.1231 Å). The ring puckering parameters are: q_2_ = 0.4198 (15) Å, q_3_ = 0.3195 (15) Å, QT = 0.5276 (14) Å and φ_2_ = 321.1 (2) ° (Cremer & Pople, 1975).

In the crystal packing, the molecules are linked through C–H···O, C–H···N and C–H···π interactions, Table 1. The short C—H···O contact, involving the trizaole-C—H and the carbonyl-O4 atoms, leads to chains along the *b* axis. These are linked along the *a* direction into a 2-D array *via* C—H···π interactions that occur between the methine-C—H and the ring centroid of the trizole ring. Fig. 2. The zigzag layers are stabilized by a number of weaker C–H···O interactions (Table 1) and stack along the *b* axis with the most significant interaction between them being of the type C—H···N, Fig. 3.

### Experimental

The reaction was carried out in a two neck 25 ml flask under a nitrogen atmosphere. To copper iodide (96 mg, 0.5 mmol) was added a solution of ((2*R*,3*S*,6*S*)-3-acetoxy-6-((trimethylsilyl)ethynyl)- 3,6-dihydro-2*H*-pyran-2-yl)methyl acetate (155 mg, 0.5 mmol) in 2 ml of THF, a solution of phenyl azide (71.4 mg, 0.6 mmol) in 3.5 ml of THF, and finally, drop wise, tetra-*n*-butyl ammonium fluoride (TBAF) (0.6 ml, 0.6 mmol) was added. The mixture was sonicated in an ultrasound bath for 90 minutes. The reaction mixture was then quenched with 20 ml of ammonium chloride and extracted with 3 *x* 15 ml of ethyl acetate. The organic phase was washed with 3 *x* 15 ml of water, dried with MgSO_4_ and then the solvent evaporated in a rota-vapor. The product was purified through a chromatographic column using ethyl acetate/hexane (1:3) as the eluent. Crystals were grown by slow evaporation from a solution of 15% of acetyl acetate in hexane at 293 K; *M*.pt: 379–382 K. ^1^H-NMR (CDCl_3_, p.p.m., 300 MHz): δ 7.99 (s, 1H); 7.74 (d, 2H, J = 7.8 Hz); 7.51 (m, 3H), 6.29 (m, 1H); 6.01 (d, 1H, J = 10.3 Hz); 5.61 (s, 1H); 5.35 (dd, 1H, J = 2.0 Hz, J = 7.8 Hz); 4.26 (d, 1H, J = 5.6 Hz); d, 1H, J = 2.9 Hz); 4.00 (ddd, 1H, J = 3.0 Hz, J = 5.6 Hz, J = 8.3 Hz); 2.08 (s, 6H); ^13^C (CDCl_3_, 75 MHz) δ (p.p.m.) 170.81; 170.38; 146.97; 137.05; 129.88; 129,58; 129,01; 125,96; 120,66; 120,39; 69,78; 67.67; 65.02; 63.08; 21.08; 20.87. HRMS calcd for C_18_H_19_N_3_O_5_ 357.1325. Found: 357.1328.

### Refinement

The H atoms were geometrically placed (C–H = 0.95–1.00 Å) and refined as riding with *U_iso_*(H) = 1.2*U_eq_*(C) and *U_iso_*(H) = 1.5*U_eq_*(methyl-C).

### Crystal data

**Table:**

C_18_H_19_N_3_O_5_	*F*(000) = 376
*M**_r_* = 357.36	*D*_x_ = 1.384 Mg m^−^^3^
Monoclinic, *P*2_1_	Cu *K*α radiation, λ = 1.54184 Å
Hall symbol: P 2yb	Cell parameters from 4088 reflections
*a* = 4.79932 (7) Å	θ = 2.7–74.0°
*b* = 16.6308 (2) Å	µ = 0.86 mm^−^^1^
*c* = 10.76331 (14) Å	*T* = 100 K
β = 93.225 (1)°	Prism, colourless
*V* = 857.73 (2) Å^3^	0.20 × 0.10 × 0.05 mm
*Z* = 2	

### Data collection

**Table:**

Agilent SuperNova Dual Cu at zero diffractometer with an Atlas detector	3369 independent reflections
Radiation source: fine-focus sealed tube	3304 reflections with *I* > 2σ(*I*)
graphite	*R*_int_ = 0.019
Detector resolution: 10.4041 pixels mm^-1^	θ_max_ = 74.2°, θ_min_ = 4.1°
ω scans	*h* = −5→5
Absorption correction: multi-scan (*CrysAlis PRO*; Agilent, 2010)	*k* = −20→20
*T*_min_ = 0.848, *T*_max_ = 0.959	*l* = −13→8
5784 measured reflections	

### Refinement

**Table:**

Refinement on *F*^2^	Secondary atom site location: difference Fourier map
Least-squares matrix: full	Hydrogen site location: inferred from neighbouring sites
*R*[*F*^2^ > 2σ(*F*^2^)] = 0.033	H-atom parameters constrained
*wR*(*F*^2^) = 0.084	*w* = 1/[σ^2^(*F*_o_^2^) + (0.0525*P*)^2^ + 0.1259*P*] where *P* = (*F*_o_^2^ + 2*F*_c_^2^)/3
*S* = 1.04	(Δ/σ)_max_ < 0.001
3369 reflections	Δρ_max_ = 0.14 e Å^−^^3^
237 parameters	Δρ_min_ = −0.19 e Å^−^^3^
1 restraint	Absolute structure: Flack (1983), 1591 Friedel pairs
Primary atom site location: structure-invariant direct methods	Flack parameter: −0.09 (15)

### Special details

**Table:**

Geometry. All s.u.'s (except the s.u. in the dihedral angle between two l.s. planes) are estimated using the full covariance matrix. The cell s.u.'s are taken into account individually in the estimation of s.u.'s in distances, angles and torsion angles; correlations between s.u.'s in cell parameters are only used when they are defined by crystal symmetry. An approximate (isotropic) treatment of cell s.u.'s is used for estimating s.u.'s involving l.s. planes.
Refinement. Refinement of *F*^2^ against ALL reflections. The weighted *R*-factor *wR* and goodness of fit *S* are based on *F*^2^, conventional *R*-factors *R* are based on *F*, with *F* set to zero for negative *F*^2^. The threshold expression of *F*^2^ > 2σ(*F*^2^) is used only for calculating *R*-factors(gt) *etc*. and is not relevant to the choice of reflections for refinement. *R*-factors based on *F*^2^ are statistically about twice as large as those based on *F*, and *R*- factors based on ALL data will be even larger.

### Fractional atomic coordinates and isotropic or equivalent isotropic displacement parameters (Å^2^)

**Table:**

	*x*	*y*	*z*	*U*_iso_*/*U*_eq_	
O1	−0.1199 (2)	0.49978 (6)	0.39298 (9)	0.0183 (2)	
O2	0.1131 (3)	0.41952 (8)	0.69075 (13)	0.0374 (3)	
O3	0.0424 (2)	0.54932 (7)	0.63709 (10)	0.0230 (2)	
O4	0.0156 (3)	0.75452 (8)	0.58495 (11)	0.0298 (3)	
O5	0.1489 (2)	0.70479 (7)	0.40355 (11)	0.0226 (2)	
N1	0.2553 (3)	0.37406 (8)	0.10112 (11)	0.0163 (2)	
N2	0.2724 (3)	0.44506 (8)	0.03950 (12)	0.0205 (3)	
N3	0.1016 (3)	0.49499 (8)	0.09098 (12)	0.0202 (3)	
C1	0.4329 (3)	0.30870 (9)	0.07023 (15)	0.0174 (3)	
C2	0.5395 (4)	0.30667 (10)	−0.04686 (15)	0.0231 (3)	
H2	0.4855	0.3461	−0.1074	0.028*	
C3	0.7260 (4)	0.24640 (11)	−0.07433 (16)	0.0274 (3)	
H3	0.8035	0.2451	−0.1536	0.033*	
C4	0.7999 (4)	0.18788 (10)	0.01356 (17)	0.0265 (3)	
H4	0.9275	0.1466	−0.0056	0.032*	
C5	0.6871 (4)	0.18983 (10)	0.12911 (17)	0.0278 (4)	
H5	0.7362	0.1494	0.1888	0.033*	
C6	0.5023 (3)	0.25052 (10)	0.15854 (15)	0.0232 (3)	
H6	0.4251	0.2520	0.2379	0.028*	
C7	0.0740 (3)	0.37932 (9)	0.19199 (13)	0.0177 (3)	
H7	0.0251	0.3385	0.2485	0.021*	
C8	−0.0244 (3)	0.45681 (9)	0.18467 (13)	0.0160 (3)	
C9	−0.2265 (3)	0.49831 (9)	0.26498 (13)	0.0176 (3)	
H9	−0.4020	0.4658	0.2614	0.021*	
C10	−0.3016 (3)	0.58149 (9)	0.21885 (15)	0.0192 (3)	
H10	−0.4012	0.5874	0.1406	0.023*	
C11	−0.2323 (3)	0.64660 (9)	0.28465 (14)	0.0200 (3)	
H11	−0.2926	0.6977	0.2543	0.024*	
C12	−0.0613 (3)	0.64194 (9)	0.40561 (14)	0.0193 (3)	
H12	−0.1821	0.6498	0.4774	0.023*	
C13	0.0863 (3)	0.56087 (9)	0.41504 (14)	0.0181 (3)	
H13	0.2238	0.5577	0.3488	0.022*	
C14	0.2357 (3)	0.54349 (10)	0.53946 (14)	0.0225 (3)	
H14A	0.3170	0.4888	0.5388	0.027*	
H14B	0.3896	0.5825	0.5550	0.027*	
C15	−0.0055 (3)	0.48239 (10)	0.70412 (15)	0.0247 (3)	
C16	−0.2217 (4)	0.49774 (14)	0.79520 (16)	0.0342 (4)	
H16A	−0.1650	0.4725	0.8749	0.051*	
H16B	−0.4003	0.4750	0.7636	0.051*	
H16C	−0.2422	0.5558	0.8070	0.051*	
C17	0.1610 (3)	0.75859 (9)	0.49755 (14)	0.0201 (3)	
C18	0.3763 (4)	0.82174 (10)	0.47939 (18)	0.0270 (4)	
H18A	0.3102	0.8734	0.5100	0.041*	
H18B	0.4096	0.8263	0.3907	0.041*	
H18C	0.5505	0.8069	0.5256	0.041*	

### Atomic displacement parameters (Å^2^)

**Table:**

	*U*^11^	*U*^22^	*U*^33^	*U*^12^	*U*^13^	*U*^23^
O1	0.0217 (5)	0.0174 (5)	0.0160 (5)	−0.0037 (4)	0.0037 (4)	−0.0012 (4)
O2	0.0421 (8)	0.0277 (7)	0.0423 (8)	−0.0015 (6)	0.0020 (6)	0.0073 (6)
O3	0.0284 (6)	0.0250 (6)	0.0162 (5)	−0.0015 (5)	0.0055 (4)	−0.0001 (4)
O4	0.0357 (7)	0.0314 (7)	0.0232 (6)	−0.0047 (5)	0.0090 (5)	−0.0096 (5)
O5	0.0253 (6)	0.0199 (5)	0.0236 (6)	−0.0061 (4)	0.0104 (5)	−0.0069 (4)
N1	0.0186 (6)	0.0138 (6)	0.0165 (6)	−0.0001 (5)	0.0014 (5)	0.0012 (5)
N2	0.0269 (7)	0.0157 (6)	0.0193 (6)	0.0015 (5)	0.0053 (5)	0.0025 (5)
N3	0.0234 (6)	0.0184 (6)	0.0194 (6)	0.0005 (5)	0.0055 (5)	0.0002 (5)
C1	0.0170 (7)	0.0150 (6)	0.0203 (7)	−0.0005 (6)	0.0013 (5)	−0.0042 (6)
C2	0.0259 (8)	0.0236 (7)	0.0201 (8)	0.0013 (6)	0.0037 (6)	−0.0024 (6)
C3	0.0283 (8)	0.0291 (9)	0.0253 (8)	0.0004 (7)	0.0064 (6)	−0.0080 (7)
C4	0.0239 (8)	0.0201 (8)	0.0355 (9)	0.0033 (6)	0.0016 (7)	−0.0088 (7)
C5	0.0312 (9)	0.0201 (8)	0.0319 (9)	0.0042 (7)	−0.0014 (7)	0.0011 (7)
C6	0.0266 (8)	0.0203 (7)	0.0228 (7)	0.0027 (7)	0.0027 (6)	0.0007 (6)
C7	0.0182 (7)	0.0176 (7)	0.0174 (7)	−0.0019 (6)	0.0029 (5)	0.0005 (6)
C8	0.0162 (7)	0.0162 (7)	0.0156 (7)	−0.0027 (5)	0.0010 (5)	−0.0019 (5)
C9	0.0172 (7)	0.0180 (7)	0.0177 (7)	−0.0022 (6)	0.0025 (5)	−0.0017 (6)
C10	0.0161 (7)	0.0209 (8)	0.0210 (7)	0.0008 (5)	0.0033 (5)	0.0009 (6)
C11	0.0189 (7)	0.0186 (7)	0.0233 (8)	0.0015 (6)	0.0074 (6)	0.0012 (6)
C12	0.0195 (7)	0.0173 (7)	0.0219 (8)	−0.0037 (6)	0.0078 (6)	−0.0030 (6)
C13	0.0180 (7)	0.0190 (7)	0.0178 (7)	−0.0037 (6)	0.0057 (5)	−0.0021 (5)
C14	0.0210 (7)	0.0280 (8)	0.0188 (7)	−0.0011 (6)	0.0045 (6)	−0.0012 (6)
C15	0.0238 (8)	0.0304 (9)	0.0195 (7)	−0.0080 (7)	−0.0032 (6)	0.0028 (6)
C16	0.0289 (9)	0.0513 (12)	0.0226 (8)	−0.0084 (9)	0.0036 (7)	0.0073 (8)
C17	0.0205 (7)	0.0174 (7)	0.0221 (7)	0.0028 (6)	−0.0005 (6)	−0.0031 (6)
C18	0.0266 (8)	0.0196 (8)	0.0349 (9)	−0.0029 (6)	0.0016 (7)	−0.0030 (6)

### Geometric parameters (Å, °)

**Table:**

O1—C13	1.4289 (17)	C7—C8	1.373 (2)
O1—C9	1.4425 (17)	C7—H7	0.9500
O2—C15	1.203 (2)	C8—C9	1.503 (2)
O3—C15	1.353 (2)	C9—C10	1.507 (2)
O3—C14	1.4435 (18)	C9—H9	1.0000
O4—C17	1.204 (2)	C10—C11	1.326 (2)
O5—C17	1.3494 (19)	C10—H10	0.9500
O5—C12	1.4537 (18)	C11—C12	1.501 (2)
N1—C7	1.3482 (19)	C11—H11	0.9500
N1—N2	1.3591 (18)	C12—C13	1.524 (2)
N1—C1	1.4320 (19)	C12—H12	1.0000
N2—N3	1.3113 (19)	C13—C14	1.511 (2)
N3—C8	1.3617 (19)	C13—H13	1.0000
C1—C6	1.384 (2)	C14—H14A	0.9900
C1—C2	1.387 (2)	C14—H14B	0.9900
C2—C3	1.387 (2)	C15—C16	1.489 (2)
C2—H2	0.9500	C16—H16A	0.9800
C3—C4	1.389 (3)	C16—H16B	0.9800
C3—H3	0.9500	C16—H16C	0.9800
C4—C5	1.384 (3)	C17—C18	1.494 (2)
C4—H4	0.9500	C18—H18A	0.9800
C5—C6	1.392 (2)	C18—H18B	0.9800
C5—H5	0.9500	C18—H18C	0.9800
C6—H6	0.9500		
			
C13—O1—C9	112.08 (11)	C9—C10—H10	119.1
C15—O3—C14	117.93 (13)	C10—C11—C12	121.97 (14)
C17—O5—C12	117.77 (12)	C10—C11—H11	119.0
C7—N1—N2	110.90 (12)	C12—C11—H11	119.0
C7—N1—C1	129.40 (13)	O5—C12—C11	107.19 (12)
N2—N1—C1	119.55 (12)	O5—C12—C13	108.46 (12)
N3—N2—N1	106.72 (12)	C11—C12—C13	109.44 (12)
N2—N3—C8	109.38 (13)	O5—C12—H12	110.6
C6—C1—C2	121.36 (14)	C11—C12—H12	110.6
C6—C1—N1	119.64 (14)	C13—C12—H12	110.6
C2—C1—N1	118.96 (14)	O1—C13—C14	107.51 (12)
C3—C2—C1	119.13 (15)	O1—C13—C12	107.63 (12)
C3—C2—H2	120.4	C14—C13—C12	115.09 (13)
C1—C2—H2	120.4	O1—C13—H13	108.8
C2—C3—C4	120.28 (15)	C14—C13—H13	108.8
C2—C3—H3	119.9	C12—C13—H13	108.8
C4—C3—H3	119.9	O3—C14—C13	109.89 (12)
C5—C4—C3	119.84 (15)	O3—C14—H14A	109.7
C5—C4—H4	120.1	C13—C14—H14A	109.7
C3—C4—H4	120.1	O3—C14—H14B	109.7
C4—C5—C6	120.51 (16)	C13—C14—H14B	109.7
C4—C5—H5	119.7	H14A—C14—H14B	108.2
C6—C5—H5	119.7	O2—C15—O3	123.73 (16)
C1—C6—C5	118.85 (15)	O2—C15—C16	125.44 (17)
C1—C6—H6	120.6	O3—C15—C16	110.83 (16)
C5—C6—H6	120.6	C15—C16—H16A	109.5
N1—C7—C8	104.66 (13)	C15—C16—H16B	109.5
N1—C7—H7	127.7	H16A—C16—H16B	109.5
C8—C7—H7	127.7	C15—C16—H16C	109.5
N3—C8—C7	108.34 (13)	H16A—C16—H16C	109.5
N3—C8—C9	122.66 (13)	H16B—C16—H16C	109.5
C7—C8—C9	128.96 (14)	O4—C17—O5	123.09 (14)
O1—C9—C8	110.58 (12)	O4—C17—C18	125.26 (14)
O1—C9—C10	111.38 (12)	O5—C17—C18	111.64 (14)
C8—C9—C10	112.47 (12)	C17—C18—H18A	109.5
O1—C9—H9	107.4	C17—C18—H18B	109.5
C8—C9—H9	107.4	H18A—C18—H18B	109.5
C10—C9—H9	107.4	C17—C18—H18C	109.5
C11—C10—C9	121.71 (14)	H18A—C18—H18C	109.5
C11—C10—H10	119.1	H18B—C18—H18C	109.5
			
C7—N1—N2—N3	−0.21 (17)	C7—C8—C9—O1	60.2 (2)
C1—N1—N2—N3	−176.19 (13)	N3—C8—C9—C10	7.9 (2)
N1—N2—N3—C8	−0.02 (16)	C7—C8—C9—C10	−174.61 (14)
C7—N1—C1—C6	−21.1 (2)	O1—C9—C10—C11	9.9 (2)
N2—N1—C1—C6	154.08 (15)	C8—C9—C10—C11	−114.87 (16)
C7—N1—C1—C2	161.09 (15)	C9—C10—C11—C12	3.5 (2)
N2—N1—C1—C2	−23.8 (2)	C17—O5—C12—C11	124.32 (14)
C6—C1—C2—C3	−2.0 (2)	C17—O5—C12—C13	−117.60 (14)
N1—C1—C2—C3	175.82 (14)	C10—C11—C12—O5	135.30 (15)
C1—C2—C3—C4	1.4 (2)	C10—C11—C12—C13	17.9 (2)
C2—C3—C4—C5	−0.1 (3)	C9—O1—C13—C14	−165.40 (12)
C3—C4—C5—C6	−0.7 (3)	C9—O1—C13—C12	70.07 (14)
C2—C1—C6—C5	1.2 (2)	O5—C12—C13—O1	−169.07 (11)
N1—C1—C6—C5	−176.58 (15)	C11—C12—C13—O1	−52.44 (15)
C4—C5—C6—C1	0.2 (3)	O5—C12—C13—C14	71.10 (15)
N2—N1—C7—C8	0.34 (16)	C11—C12—C13—C14	−172.27 (13)
C1—N1—C7—C8	175.81 (14)	C15—O3—C14—C13	117.54 (15)
N2—N3—C8—C7	0.23 (17)	O1—C13—C14—O3	−63.44 (16)
N2—N3—C8—C9	178.14 (13)	C12—C13—C14—O3	56.45 (17)
N1—C7—C8—N3	−0.34 (16)	C14—O3—C15—O2	3.6 (2)
N1—C7—C8—C9	−178.08 (14)	C14—O3—C15—C16	−176.58 (13)
C13—O1—C9—C8	78.46 (14)	C12—O5—C17—O4	3.6 (2)
C13—O1—C9—C10	−47.37 (15)	C12—O5—C17—C18	−177.05 (14)
N3—C8—C9—O1	−117.28 (15)		

### Hydrogen-bond geometry (Å, °)

**Table:**

*D*—H···*A*	*D*—H	H···*A*	*D*···*A*	*D*—H···*A*
C7—H7···O4^i^	0.95	2.29	3.2207 (19)	167
C9—H9···Cg1^ii^	1.00	2.68	3.5362 (16)	144
C16—H16a···N3^iii^	0.98	2.62	3.463 (2)	145
C16—H16b···O2^ii^	0.98	2.59	3.570 (2)	177
C18—H18a···O1^iv^	0.98	2.54	3.516 (2)	174
C18—H18c···O4^v^	0.98	2.45	3.400 (2)	164

Symmetry codes: (i) −*x*, *y*−1/2, −*z*+1; (ii) *x*−1, *y*, *z*; (iii) *x*, *y*, *z*+1; (iv) −*x*, *y*+1/2, −*z*+1; (v) *x*+1, *y*, *z*.
